# Glutathione and Its Biosynthetic Intermediates Alleviate Cesium Stress in Arabidopsis

**DOI:** 10.3389/fpls.2019.01711

**Published:** 2020-01-21

**Authors:** Eri Adams, Takae Miyazaki, Shunsuke Watanabe, Naoko Ohkama-Ohtsu, Mitsunori Seo, Ryoung Shin

**Affiliations:** ^1^ RIKEN Center for Sustainable Resource Science, Yokohama, Japan; ^2^ Institute of Agriculture, Tokyo University of Agriculture and Technology (TUAT), Fuchu, Japan; ^3^ Institute of Global Innovation Research, Tokyo University of Agriculture and Technology (TUAT), Fuchu, Japan

**Keywords:** *Arabidopsis thaliana*, cesium, glutathione, jasmonates, potassium, phytoremediation, sodium, sulphur supply

## Abstract

Phytoremediation is optimized when plants grow vigorously while accumulating the contaminant of interest. Here we show that sulphur supply alleviates aerial chlorosis and growth retardation caused by cesium stress without reducing cesium accumulation in *Arabidopsis thaliana*. This alleviation was not due to recovery of cesium-induced potassium decrease in plant tissues. Sulphur supply also alleviated sodium stress but not potassium deficiency stress. Cesium-induced root growth inhibition has previously been demonstrated as being mediated through jasmonate biosynthesis and signalling but it was found that sulphur supply did not decrease the levels of jasmonate accumulation or jasmonate-responsive transcripts. Instead, induction of a glutathione synthetase gene *GSH2* and reduction of a phytochelatin synthase gene *PCS1* as well as increased accumulation of glutathione and cysteine were observed in response to cesium. Exogenous application of glutathione or concomitant treatments of its biosynthetic intermediates indeed alleviated cesium stress. Interestingly, concomitant treatments of glutathione biosynthetic intermediates together with a glutathione biosynthesis inhibitor did not cancel the alleviatory effects against cesium suggesting the existence of a glutathione-independent pathway. Taken together, our findings demonstrate that plants exposed to cesium increase glutathione accumulation to alleviate the deleterious effects of cesium and that exogenous application of sulphur-containing compounds promotes this innate process.

## Introduction

Plants readily absorb cesium when present in the soil mainly through the potassium uptake system in the roots. Potassium is an essential nutrient for plants, a lack of which causes severe growth retardation while cesium has no known beneficial role in plants and its accumulation in the plants inhibits growth. Due to the physicochemical similarities among the alkali metals, cesium and potassium share the same uptake system and cesium competes with potassium at the root surface and disrupts the physiological roles of potassium inside the plant cells (White and Broadley, 2000). We have recently shown that cesium selectively inhibits potassium intake through a potassium channel complex AKT1-KC1 and the inability of cesium-treated plants to accumulate potassium is the major cause of growth retardation in *Arabidopsis thaliana* (Adams et al., 2018). Plants exposed to cesium induce expression of a potassium transporter gene (*HAK5*), a potassium deficiency marker gene, and activate jasmonate (JA) biosynthesis and signalling (Adams et al., 2013). Cesium also inactivates proteins by interacting with potassium binding sites and changes a wide variety of gene expression and microRNA processing (Hampton et al., 2004; Sahr et al., 2005a; Sahr et al., 2005b; Jung et al., 2015). Although the natural occurrence of cesium in soils is relatively low, occasional inadvertent release of cesium, most likely radiocesium, through anthropogenic activities such as the nuclear power plant failure at Chernobyl and Fukushima can seriously contaminate soils. Radioactive cesium in the soil is not only harmful for the environment *per se*, but it can also enter and be concentrated in the human food chain *via* plants.

Phytoremediation is a biological remediation method to remove toxic compounds from contaminated land and is considered to be more cost-effective and environmentally-friendly than conventional physical and chemical remediation methods. As phytoremediation utilizes the ability of plants to absorb the metal of interests, it is non-destructive to the soil structure and is particularly preferred in farmland. Plants that accumulated the contaminant are harvested and subject to desiccation and combustion so that the volume of radioactive waste is dramatically reduced compared to the volume involved when physical removal of contaminated soil is deployed (Dushenkov, 2003). Ideal phytoremediation is enabled when plants 1) accumulate a large amount of the contaminant in the aerial parts and 2) maintain vigorous growth while accumulating the contaminant. For phytoremediation of cesium, a wide variety of plant species have been investigated in the search for a “hyperaccumulator” and biological or chemical factors which improve uptake ability of plants have been tested (Burger and Lichtscheidl, 2018). Our previous screening effort isolated a chemical compound, methyl cysteinate, which promotes cesium accumulation in plants (Adams et al., 2017). However, plants accumulating high levels of cesium suffer stunted growth. It has been known that a plentiful supply of potassium mitigates cesium-induced growth inhibition but only in exchange for reduced cesium accumulation (Zhu and Smolders, 2000). While the addition of potassium helps to protect agricultural crops from being contaminated with radiocesium, the addition of sulphur resulted in alleviation of growth inhibition by cesium without reduction of cesium accumulation in plants.

The involvement of sulphur metabolism in metal stress response has been well reported (Seth et al., 2012; Anjum et al., 2015). A sulphur-containing metabolite, glutathione (GSH), acts as a strong non-protein antioxidant that protects cells suffering from metal stress which causes oxidative damage (Noctor et al., 2011). GSH and downstream metabolites, phytochelatins (PCs), also function as metal chelators which then sequestrate the metal into vacuoles upon binding, a common detoxification strategy for heavy metals (Hasanuzzaman et al., 2017). Some examples of the interaction between metal stress and the sulphur metabolic pathway in plants are introduced here. Cadmium exposure in Arabidopsis induces expression of genes encoding sulphate transporter (SULTR), serine acetyltransferase (SERAT), γ-glutamylcysteine (γ-Glu-Cys) synthetase (GSH1), and GSH synthetase (GSH2) and in turn increases sulphate uptake and accumulation of cysteine (Cys) and PCs (Xiang and Oliver, 1998; Howarth et al., 2003; Yamaguchi et al., 2016; Ferri et al., 2017). Chromium has been shown to induce PC synthase genes (*PCS1* and *PCS2*) and increase hydrogen sulfide, Cys and GSH levels, while copper induces *GSH1* and *GSH2* in Arabidopsis (Xiang and Oliver, 1998; Fang et al., 2016). Proteomic analysis has revealed that GSH, PC and glucosinolate biosynthesis pathways are up-regulated during a response to lead in Arabidopsis (Zhu et al., 2016). Toxic levels of selenium have been demonstrated to induce a series of genes encoding SULTRs and sulphur metabolism enzymes in a selenium-resistant Arabidopsis accession Col-0 but not in a selenium-sensitive accession Ws-2 (Tamaoki et al., 2008). Meanwhile, a light metal, sodium, has also been shown to induce a range of *SULTR*s and sulphur metabolism genes in Arabidopsis (Cao et al., 2014). In mustard (*Brassica juncea*), sulphur supply has been indicated to alleviate sodium stress through increased GSH accumulation, promoted photosynthesis and reduced ethylene production (Fatma et al., 2014; Nazar et al., 2014).

In the present study, we demonstrated that sulphur supply alleviated the deleterious effects of cesium without reducing cesium accumulation in Arabidopsis. This alleviation was not due to increased potassium accumulation or reduced JA production. This alleviation was also not due to well-known metal detoxifying agents, PCs. Instead, we describe that GSH and its biosynthetic intermediates are important for retrieving growth in plants suffering from cesium stress.

## Material and Methods

### Plant Material and Growth Condition

The *A. thaliana* (L.) Heynh. accession Col-0 was used as a wild type. The mutant seeds *sultr1;1* (salk_093256c), *sultr1;2* (salk_122974), *sultr1;3* (salk_018910), *sultr2;1* (salk_109907c), *sultr2;2* (salk_111268c), *sultr3;1* (salk_127024c), *sultr3;2* (salk_023980c), *sultr3;3* (salk_000822c), *sultr3;4* (salk_100362), *sultr3;5* (salk_128559), *sultr4;1* (salk_103873c), *sultr4;2* (salk_103827c), and *cad1*-3 (CS68125) were obtained from the ABRC (Arabidopsis Biological Resource Center, https://abrc.osu.edu/) and *aos* (CS6149) and *jar1*-1 (CS8072) were obtained from the NASC (Nottingham Arabidopsis Stock Centre, http://nasc.nott.ac.uk). Seeds were surface-sterilized with 70% (v/v) ethanol and 0.05% (v/v) Triton X-100 and sown on media containing 2 mM Ca(NO_3_)_2_, 0.5 mM phosphoric acid, 0.75 mM MgSO_4_, 50 μM H_3_BO_3_, 10 μM MnCl_2_, 2 μM ZnSO_4_, 1.5 μM CuSO_4_, 0.075 μM NH_4_Mo_7_O_24_, and 74 μM Fe-EDTA, pH 5.8 with Ca(OH)_2_, 1% (w/v) sucrose and 1% (w/v) of SeaKem agarose (Lonza) or agarose L03 (TaKaRa) supplemented with designated concentrations of KCl, CsCl and other chemicals. For sulphur supply, CaSO_4_ was supplemented in addition to 0.75 mM MgSO_4_ in the base media to add up to the indicated concentrations. After stratification for three to four days at 4°C, plants were placed in a growth cabinet at 22°C in a 16 h light/8 h dark photocycle with a light intensity of 70–90 μmol m^−2^ s^−1^.

### Phenotype Quantification

Aerial phenotype of cesium-treated plants was scored as follows: seedlings with fully expanded green aerial parts were given score 2, those with more than half the number of leaves (cotyledons plus true leaves) showing chlorosis were given score 0 and those with intermediate phenotype given score 1. Primary root lengths were measured on images using ImageJ (Schneider et al., 2012).

### qRT-PCR Analysis

Seedlings grown for eight days on media containing 1.75, 0.5 or 0.01 mM KCl with or without 0.3 mM CsCl and additional CaSO_4_ were divided into roots and shoots, flash-frozen in liquid N_2_ and ground using a mixer mill. Total RNA was extracted, treated with DNaseI (Invitrogen) and synthesized into cDNA using SuperScript III (Invitrogen). Quantitative real-time reverse transcription-PCR (qRT-PCR) was performed using THUNDERBIRD SYBR qPCR mix (TOYOBO) and a Mx3000P qPCR system (Agilent Technologies). The amplification conditions were 95°C for 15 s and 60°C for 30 s. The cycle was repeated 40 times, preceded by 95°C for 1 min and followed by a dissociation programme to create melting curves. Three biological replicates each of which contains >30 seedlings were analyzed. The β-tubulin gene (*TUB2*, At5g62690) was used as a reference gene. The primer sequences for *HAK5*, *VSP2*, *PDF1.2*, *SULTR1;1*, *SULTR1;2*, *SULTR3;1*, *SULTR3;2*, *SULTR3;3*, *SULTR3;4,* and *SULTR3;5* are shown in the previous publications (Adams et al., 2013; Cao et al., 2014; Ferri et al., 2017). The rest of the primers used are listed in **Supplementary Table S1**.

### Elemental Analysis

Whole seedlings were washed in Milli-Q water, dried on a paper towel, placed in a paper envelope and dried in an oven at 65°C for three to four days. Typically, 20 to 60 seedlings were pooled as one biological replicate and three biological replicates were analyzed. Approximately 2 mg of dried samples were extracted in 1 ml of 60% (v/v) HNO_3_ at 125°C for 1 h, followed by 1 ml of 30% (v/v) H_2_O_2_ and diluted with Milli-Q water to get a total volume of 10 ml. For potassium and sodium analysis, samples were further diluted 10 and 100 times with 6% (v/v) HNO_3_. For cesium analysis, 0.1% (w/v) KCl was added to each sample and standard solution to prevent ionisation of cesium, according to the manufacturer’s instructions (PerkinElmer). Potassium, sodium, and cesium concentrations were measured on a flame atomic absorption spectrometer AAnalyst 200 (PerkinElmer). Concentrations were calculated against each standard curve.

### Glutathione and Cysteine Quantification

Whole seedlings were harvested and fresh weights of approximately 20 to 40 seedlings were determined. Flash-frozen samples were ground using a mixer mill, extracted with 10 mM HCl and derivatized with monobromobimane as described previously (Minocha et al., 2008; Nishida et al., 2016), followed by HPLC (Shimadzu Corp.) with a Shim-pac FC-ODS column (150 × 4.6 mm, Shimadzu Corp.). The standard chemicals were purchased from Wako Pure Chemical Industries Ltd.

### JA Quantification

Quantification of jasmonic acid (JA) and jasmonoyl–isoleucine (JA-Ile) were performed as described previously (Kanno et al., 2016). Briefly, whole seedlings grown in each condition were freeze-dried and then homogenized in the extraction solution [80% (v/v) acetonitrile containing 1% (v/v) acetic acid] with defined amounts of isotopically labelled JA (D_2_-JA) and JA-Ile (^13^C_6_-JA-Ile) as internal standards. After incubation for 12 h at 4°C, the homogenate was centrifuged at 3,000 × g for 20 min. The supernatants were dried using nitrogen gas and dissolved with 1% (v/v) acetic acid solution. After purification of hormones by solid-phase extraction columns (Oasis WAX, Waters), extracts were dried using nitrogen gas and dissolved in 1% (v/v) acetic acid solution. Endogenous hormone levels were determined using a UPLC-MS/MS system consisting of a quadrupole/time-of-flight tandem mass spectrometer (Triple TOF 5600, AB SCIEX) and a Nexera UPLC system (Shimadzu Corp.) as described previously. To calculate plant hormone concentrations from the LC–MS–MS data, a software MultiQuant 2.0 (AB SCIEX) was used.

### Statistical Analysis

All statistical analysis were performed using Prism (GraphPad Software). Green score was analyzed with Kruskal-Wallis test with Dunn’s multiple comparison posttest using Prism (GraphPad Software) to determine the statistical significance. T-test was performed in order to determine the significant difference of *VSP2* and *PDF1.2* expression in Col-0 grown on media containing 1.75 mM K and 2 mM S compared to control condition (0.75 mM S). All other cases, one-way ANOVA tests with Bonferroni’s or Dunnett’s multiple comparison tests were used in order to determine the statistical significance.

## Results

### Sulphur Supply Alleviates Cesium Stress in Plants

**Figure 1:**
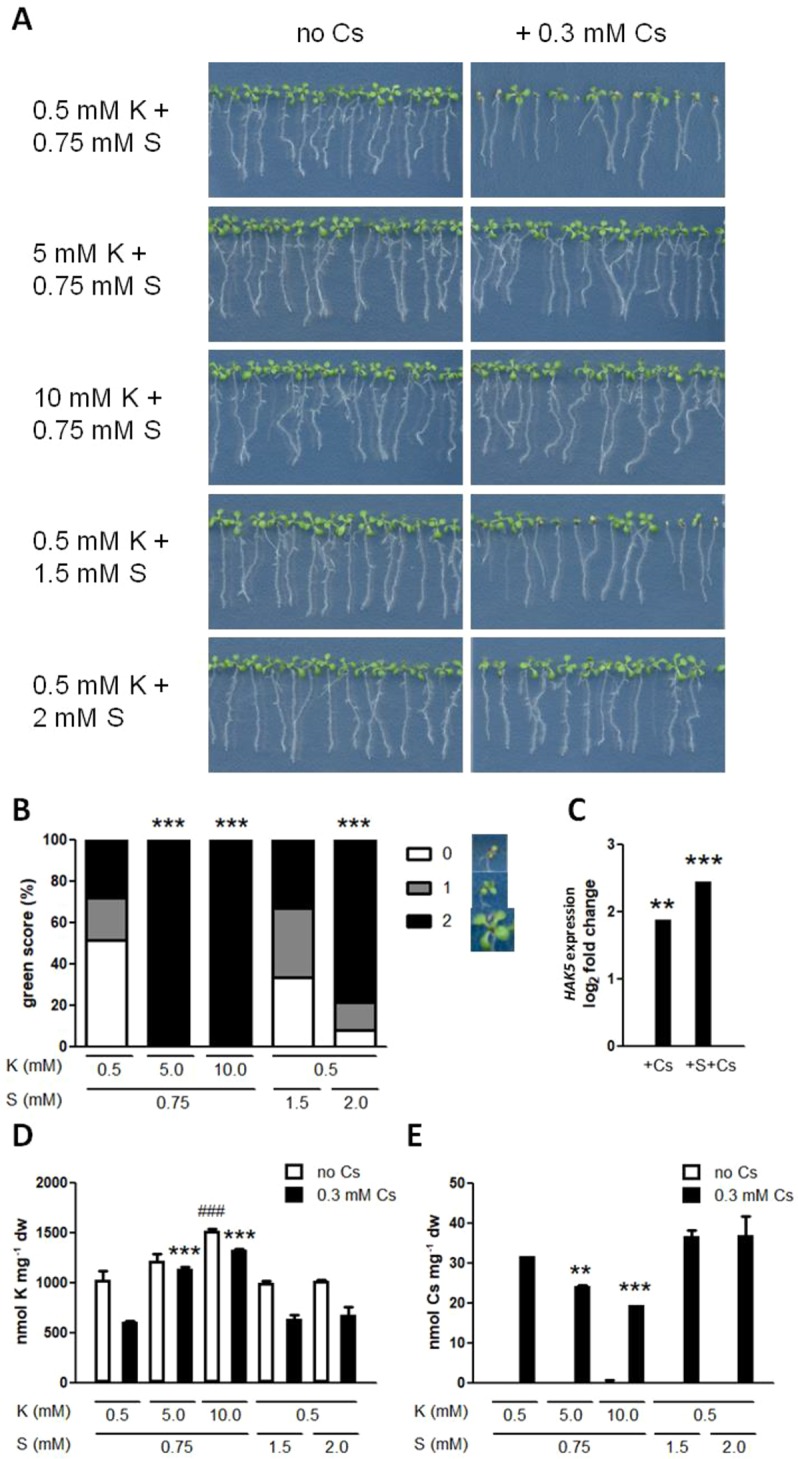
Effects of potassium and sulphur supply on cesium stress. **(A)** Eight-day-old wild type (Col-0) seedlings germinated on media containing indicated concentrations of potassium (K) and sulphate (S) with or without cesium (Cs). **(B)** Percentile green scores for cesium-treated seedlings derived from **(A)**. Statistically significant differences relative to the control cesium condition (0.5 mM K + 0.75 mM S + 0.3 mM Cs) were determined by Kruskal-Wallis test with Dunn’s multiple comparison posttest (n > 38, *P* < 0.001). Examples of a seedling with each score are provided next to the score legend. **(C)** Expression of *HAK5* in Col-0 grown on media containing 1.75 mM K and 0.3 mM Cs with or without 2 mM S for eight days. Values are log_2_ ratios relative to expression in the control seedlings grown in the absence of cesium (1.75 mM K + 0.75 mM S). Statistically significant differences were determined by one-way ANOVA with Bonferroni’s multiple comparison posttest (n = 3, ** for *P* < 0.01, *** for *P* < 0.001). **(D)** K and **(E)** Cs concentrations in seedlings derived from **(A)**. Statistically significant differences were determined by one-way ANOVA with Bonferroni’s multiple comparison posttest (n = 3, ^###^ for *P* < 0.001 relative to the non-cesium control, ** for *P* < 0.01, *** for *P* < 0.001 relative to the cesium-treated control) and error bars represent the SE.

In order to assess the effects of nutrient supply on plants experiencing cesium stress, a plentiful supply of either potassium or sulphur in the form of sulphate was applied to Arabidopsis wild type (Col-0) seedlings. Metal stress usually causes uniform growth inhibition response but with cesium the response is often variable. At a sub-optimal potassium (0.5 mM KCl) with basal sulphur (0.75 mM MgSO_4_) condition, seedlings supplied with cesium (0.3 mM CsCl) showed a characteristic mixed phenotype with some suffering aerial chlorosis and reduced root growth and others only minor growth retardation (**Figure 1A**). The similar phenotype was found in hydroponically grown seedlings. To highlight and quantify this distinctive aerial phenotype caused by cesium, green scoring was introduced and each seedling was given score 0–2 with score 0 being the severest cesium phenotype (see the *Material and Methods* section for the detailed scoring scale, **Figure 1B**). Increasing potassium concentrations to 5 or 10 mM totally cancelled the visible cesium phenotype (**Figures 1A, B**). Increasing the sulphur concentration to 2 mM dramatically improved the health and growth of plants exposed to cesium, however it did not alter cesium-induced *HAK5* expression (*P* > 0.05, **Figures 1A**–**C**). *HAK5* expression was not altered by sulphur supply in the absence of cesium either (0.77 times expression relative to the control, *P* > 0.05). Accordingly, potassium concentrations in the plants were not increased by sulphur supply in the presence or absence of cesium (**Figure 1D**). Moreover, sulphur supply did not alter cesium accumulation unlike potassium supply which resulted in a dose-dependent reduction of cesium concentrations in the plants (**Figure 1E**).

### Sulphur Supply Alleviates Sodium Stress but Not Potassium Deficiency Response

**Figure 2:**
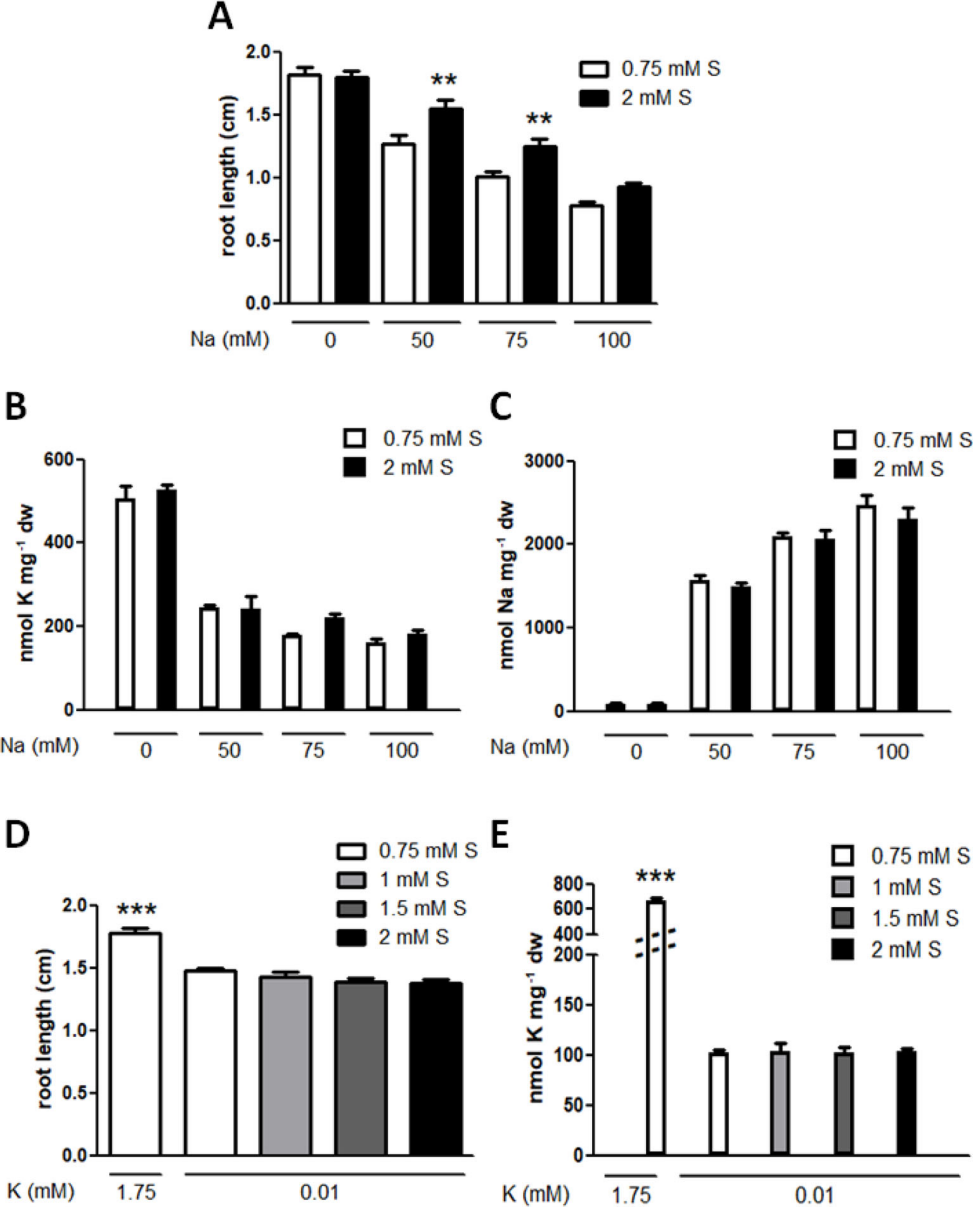
Effects of sulphur supply on sodium stress and potassium deficiency response. **(A)** Root lengths of the wild type (Col-0) grown on media containing optimal potassium (1.75 mM K) and the indicated concentrations of sodium (Na) and sulphate (S) for eight days. Statistically significant differences between the basal (0.75 mM) and abundant (2 mM) S treatments at each Na concentration were determined by one-way ANOVA with Bonferroni’s multiple comparison posttest and marked with asterisks (n > 30, *P* < 0.01). Error bars represent the SE. **(B)** K and **(C)** Na concentrations in seedlings derived from **(A)**. No statistical difference between the basal and abundant S treatments at each Na concentration was determined by one-way ANOVA with Bonferroni’s multiple comparison posttest (n = 3) and error bars represent the SE. **(D)** Root lengths and **(E)** K concentrations of Col-0 grown in the optimal K condition or K deficiency (10 μM) with the indicated concentrations of S for eight days. A statistically significant difference relative to those treated with potassium deficiency and the basal S condition was determined by one-way ANOVA with Bonferroni’s multiple comparison posttest and marked with asterisks (n > 32 for root lengths, n = 3 for K concentrations, *P* < 0.001). Error bars represent the SE.

Being an alkali metal, sodium is also known to compete with potassium and initiates a potassium deficiency response in plants. Increasing concentrations of sodium inhibited root growth in a dose-dependent manner but 2 mM sulphur supply mitigated this growth inhibition (**Figure 2A**). However, this growth mitigation was not due to altered potassium or sodium concentrations in the plants (**Figures 2B, C**). In contrast, 1–2 mM sulphur supply could not recover potassium deficiency phenotype or the reduced levels of potassium accumulation in plants (**Figures 2D, E**).

### Sulphate Transporters Do Not Seem to be Involved in Cesium Stress Alleviation by Sulphur Supply

**Figure 3:**
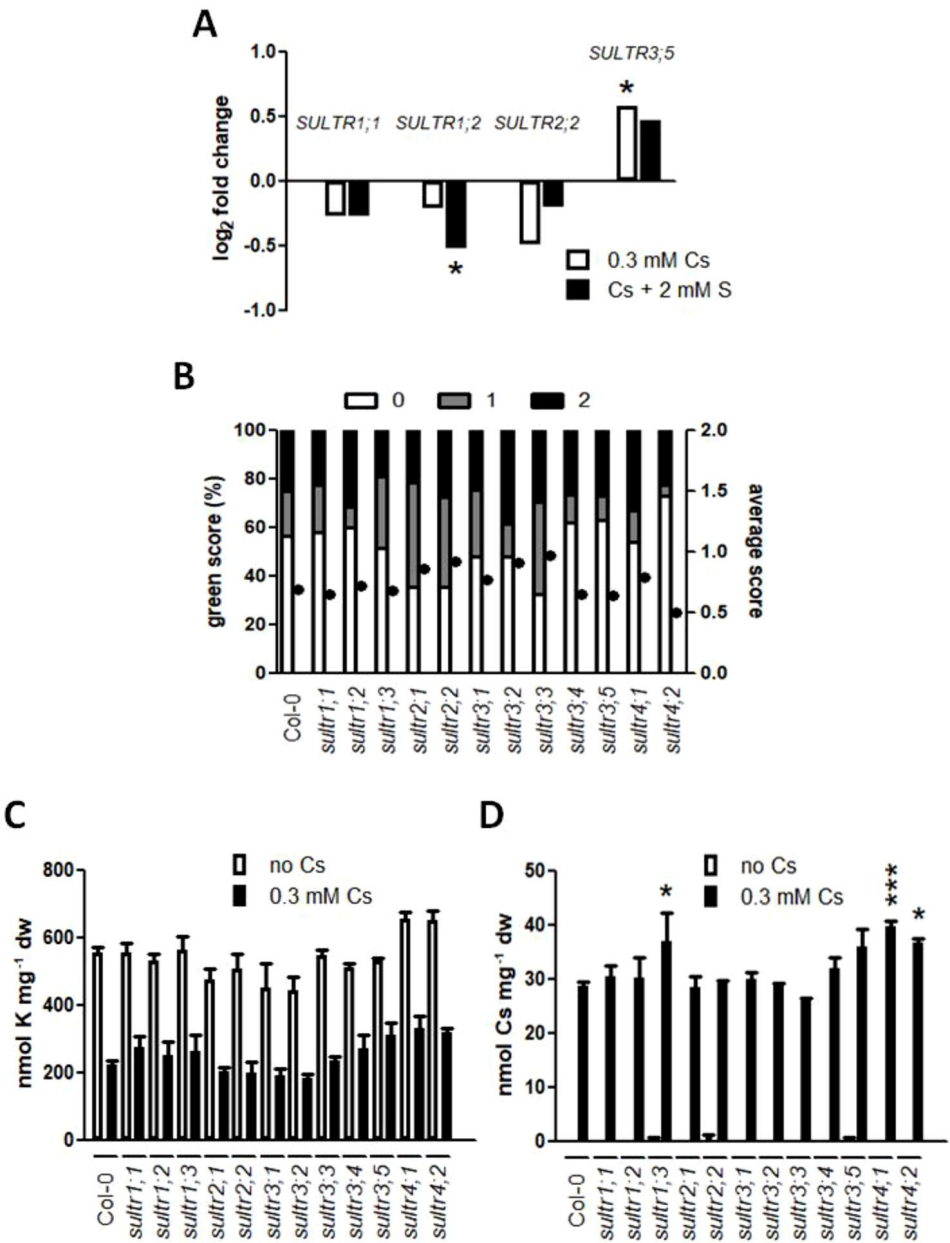
Involvement of sulphate transporters (SULTRs) in cesium response. **(A)** Expression of *SULTR1;1*, *SULTR1;2*, *SULTR2;2* and *SULTR3;5* in the roots of the wild type (Col-0) grown on media containing 1.75 mM potassium (K) and 0.3 mM cesium (Cs) with or without 2 mM sulphate (S) for eight days. Values are log_2_ ratios relative to expression in the control seedlings grown in the absence of cesium (1.75 mM K + 0.75 mM S). Statistically significant differences were determined by one-way ANOVA with Bonferroni’s multiple comparison posttest and marked with asterisks (n = 3, *P* < 0.05). **(B)** Percentile green scores (left axis) and average scores (right axis, closed circles) for *sultr* mutants treated with 0.5 mM K, 0.75 mM S and 0.3 mM Cs for eight days. No statistical difference relative to Col-0 was determined by Kruskal-Wallis test with Dunn’s multiple comparison posttest (n > 60). **(C)** K and **(D)** Cs concentrations in seedlings derived from **(B)**. Statistically significant differences were determined by one-way ANOVA with Bonferroni’s multiple comparison posttest (n = 3, * for *P* < 0.05, *** for *P* < 0.001 relative to Col-0) and error bars represent the SE.

Sulphur-induced alleviation of cesium response in plants was not because sulphur could reverse a potassium accumulation loss that was caused by cesium or sodium. To test whether increased sulphur uptake and translocation in the cells are responsible for cesium stress alleviation, gene expression of *SULTR*s was analyzed. Expression of genes encoding high-affinity sulphate transporters, SULTR1;1 and SULTR1;2, or that of a gene encoding a low-affinity sulphate transporter, SULTR2;2 (Takahashi et al., 2000; Yoshimoto et al., 2002), was not altered in roots treated with cesium relative to those on the optimal potassium condition (1.75 mM KCl, **Figure 3A**). In contrast, expression of *SULTR3;5* responsible for the root-to-shoot sulphate transport (Kataoka et al., 2004a) was mildly induced in response to cesium. A complete expression pattern of all *SULTR*s in roots and shoots treated with cesium and sulphur supply in a sub-optimal potassium condition (0.5 mM KCl) is shown in **Supplementary Figure S1**.

To further investigate the role of SULTRs in cesium stress alleviation, insertional mutants of each *SULTR* were analyzed for cesium response and compared with the wild type. There was no statistical difference recognized in terms of green score and potassium accumulation between *sultr* mutants and the wild type (**Figures 3B, C**). Interestingly, *sultr1;3*, *sultr4;1,* and *sultr4;2* were shown to accumulate more cesium (**Figure 3D**). *sultr* mutants did not show altered phenotypic response to potassium deficiency relative to the wild type although *SULTR1;2*, *SULTR3;1,* and *SULTR3;5* were up-regulated in roots under potassium deficiency (**Supplementary Figure S1**).

### GSH and Its Biosynthetic Intermediates, but Not PCs, Are Important for Cesium Stress Alleviation

**Figure 4:**
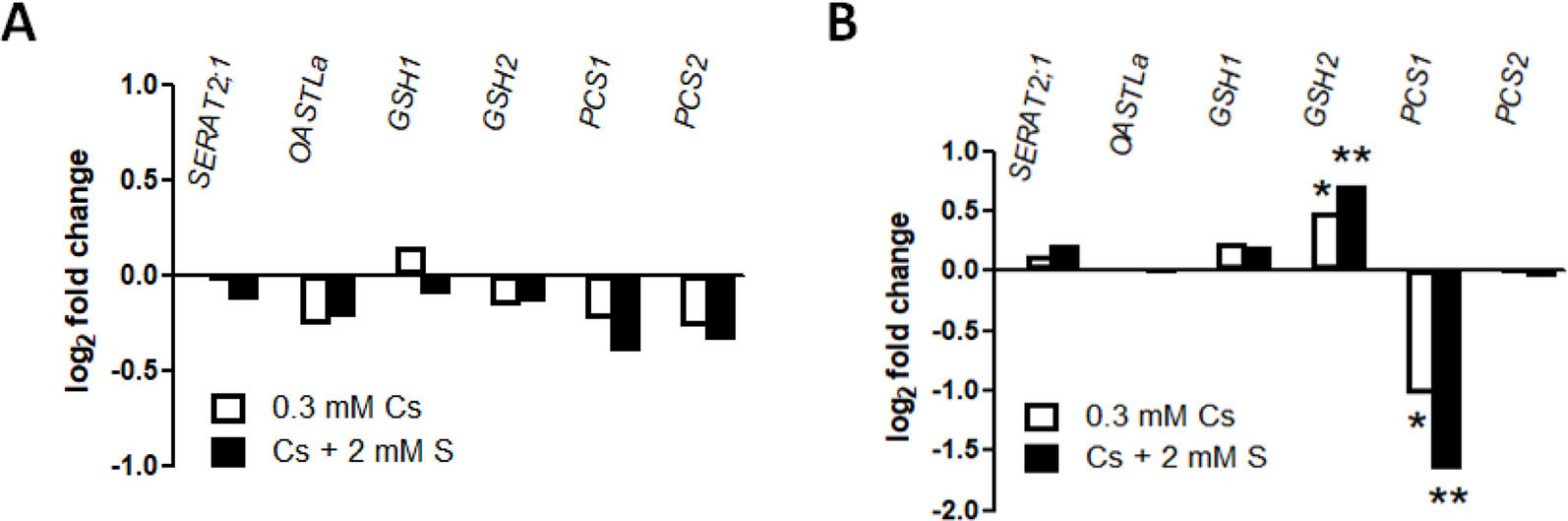
Expression of sulphur metabolism genes in response to cesium. Gene expression in roots **(A)** and shoots **(B)** of the wild type (Col-0) grown on media containing 1.75 mM potassium (K) and 0.3 mM cesium (Cs) with or without 2 mM sulphate (S) for eight days. Values are log_2_ ratios relative to expression in the control seedlings grown in the absence of cesium (1.75 mM K + 0.75 mM S). Statistically significant differences were determined by one-way ANOVA with Bonferroni’s multiple comparison posttest (n = 3, * for *P* < 0.05, ** for *P* < 0.01).

Expression of sulphate metabolism genes was analyzed to understand which forms of sulphur-containing metabolites were important for cesium stress alleviation. Sulphate, once absorbed by plants, goes through a sequential assimilative reduction reaction and is synthesized into Cys. Serine acetyltransferase (SERAT) and *O*-acetlyserine(thiol)lyase (OASTL) are the two classes of proteins involved in Cys synthesis (Hatzfeld et al., 2000; Kawashima et al., 2005). *SERAT2;1*, a gene encoding one of three major SERATs (Kawashima et al., 2005), and *OASTLa*, a gene encoding a cytosolic form of OASTL (Jost et al., 2000), were not responsive to cesium in both roots and shoots (**Figure 4**). GSH is synthesized through a sequential addition of glutamic acid (Glu) and glycine (Gly) to Cys by the function of GSH1 and GSH2, respectively (May and Leaver, 1994; Ullmann et al., 1996) while PCs are synthesized from GSH by the action of PCS1 and PCS2 (Howden et al., 1995; Cazale and Clemens, 2001). Expression analysis revealed that *GSH2* was induced and *PCS1* was reduced in response to cesium in shoots and these alterations were more profound in shoots treated with cesium together with a supply of sulphur (**Figure 4**).

**Figure 5:**
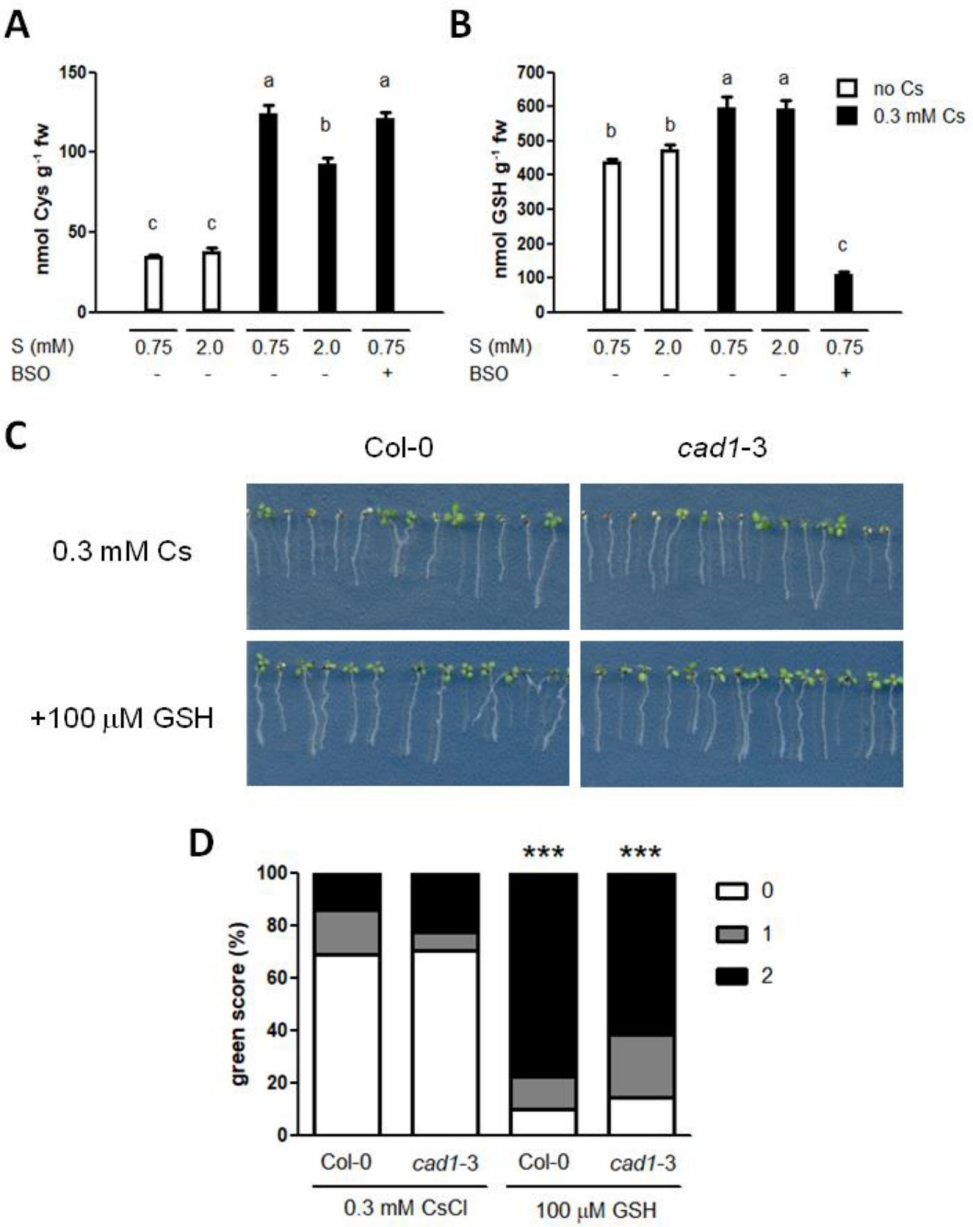
Effects of glutathione on cesium stress. **(A)** Cysteine (Cys) and **(B)** Glutathione (GSH) levels in the wild type (Col-0) germinated and grown on media containing 0.5 mM potassium (K) with or without 0.3 mM cesium (Cs), 300 μM buthionine sulphoximine (BSO) and indicated concentrations of sulphate (S) for eight days. Alphabetical letters show statistical differences (*P* < 0.01) determined by one-way ANOVA with Bonferroni’s multiple comparison posttest. **(C)** Eight-day-old Col-0 and *cad1*-3 seedlings germinated on media containing 0.5 mM K and 0.3 mM Cs with or without 100 μM GSH. **(D)** Percentile green scores for cesium-treated seedlings derived from **(C)**. Statistically significant differences relative to the control cesium condition for each line were determined by Kruskal-Wallis test with Dunn’s multiple comparison posttest and marked with asterisks (n > 40, *P* < 0.001). No statistical difference was observed between Col-0 and *cad1*-3 of the same treatment.

Next, internal levels of Cys, GSH and PCs were determined in the seedlings treated with cesium and sulphur supply. In consistency with *GSH2* expression (**Figure 4B**) and our previous results (Adams et al., 2017), cesium treatment increased both Cys and GSH but sulphur supply did not (**Figures 5A, B**). PC2 and PC3 levels were below detection limits in all the conditions tested. Exogenous application of GSH indeed alleviated aerial chlorosis caused by cesium to the degree equivalent to the sulphur supply (**Figures 5C, D**). A mutant defective in PC synthesis, *cad1*-3 (Howden et al., 1995) showed a wild type-like response to cesium and this phenotype was also recovered to the same degree as the wild type by GSH treatment in excellent agreement with the *PCS* expression patterns and PC quantification results (**Figures 4B** and **5C**, **D**).

**Figure 6:**
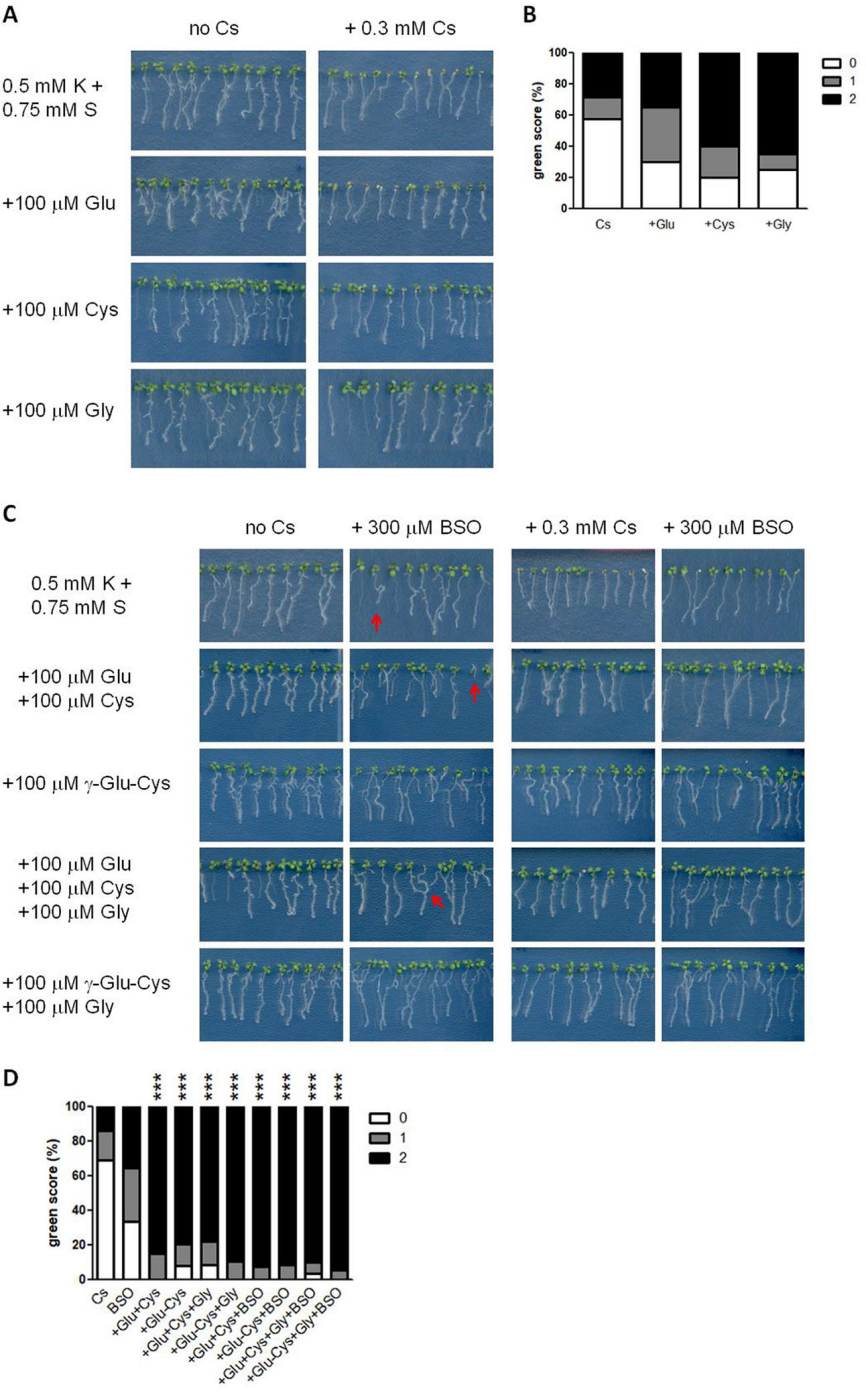
Effects of glutathione biosynthetic intermediates on cesium stress. **(A)** Eight-day-old wild type (Col-0) seedlings germinated on media containing 0.5 mM potassium (K) with or without 0.3 mM cesium (Cs) and 100 μM glutamic acid (Glu), cysteine (Cys) or glycine (Gly). **(B)** Percentile green scores for cesium-treated seedlings derived from **(A)**. No statistical difference relative to the control cesium condition (0.5 mM K + 0.75 mM S + 0.3 mM Cs) was determined by Kruskal-Wallis test with Dunn’s multiple comparison posttest (n > 20). **(C)** Eight-day-old Col-0 seedlings germinated on media containing 0.5 mM K and the indicated concentrations of Cs, Glu, Cys, Gly, γ-glutamylcysteine (γ-Glu-Cys) and buthionine sulphoximine (BSO). Red arrows indicate the distinct growth retardation phenotype the production of which is occasionally provoked by BSO treatment. **(D)** Percentile green scores for cesium-treated seedlings derived from **(C)**. Statistically significant differences relative to the control cesium condition were determined by Kruskal-Wallis test with Dunn’s multiple comparison posttest and marked with asterisks (n > 31, *P* < 0.001).

To further define the crucial sulphur-containing metabolites for cesium stress alleviation, the effects of 100 μM Glu, Cys or Gly on plant growth were investigated. Glu stunted primary root growth and increased secondary root structures both in the presence and absence of cesium (**Figure 6A**). Upon calculating green score, the percentage of the seedlings with score 0 decreased by treatment with any of these three amino acids and that with score 2 increased by Cys or Gly supplementation but these changes were not statistically significant relative to the non-amino acid supplemented control (**Figure 6B**). Next, concomitant treatments of Glu, Cys, Gly and γ-Glu-Cys were tested. Applying Glu together with Cys dramatically recovered cesium phenotype (**Figures 6C, D**) to the same degree as with sulphur supply (**Figures 1A, B**). A single application of γ-Glu-Cys stunted plant growth in general in the absence of cesium while it strongly alleviated cesium-induced aerial chlorosis (**Figures 6C, D**). Concomitant application of Glu, Cys and Gly or γ-Glu-Cys and Gly also alleviated cesium stress. Interestingly, Glu- or γ-Glu-Cys-induced growth inhibition was somehow attenuated by addition of Gly. Buthionine sulphoximine (BSO) is a specific inhibitor of GSH1 (Griffith, 1982). Three hundred μM of BSO treatment caused occasional stunting, partial loss of root gravitropism and aerial chlorosis independent of the effects caused by cesium (**Figure 6C**, indicated by red arrows) and the seedlings displaying these symptoms were excluded from green scoring. Moreover, the alleviation effects of concomitant treatments among Glu, Cys, γ-Glu-Cys, and Gly against cesium stress were not disrupted by the addition of BSO. Effectiveness of BSO was confirmed by measuring GSH levels in the seedlings treated with cesium and BSO (**Figure 5B**). Meanwhile, BSO treatment did not alter the internal Cys level (**Figure 5A**).

### Sulphur-Induced Alleviation of Cesium Stress Is Independent of JA Biosynthesis and Signalling

**Figure 7:**
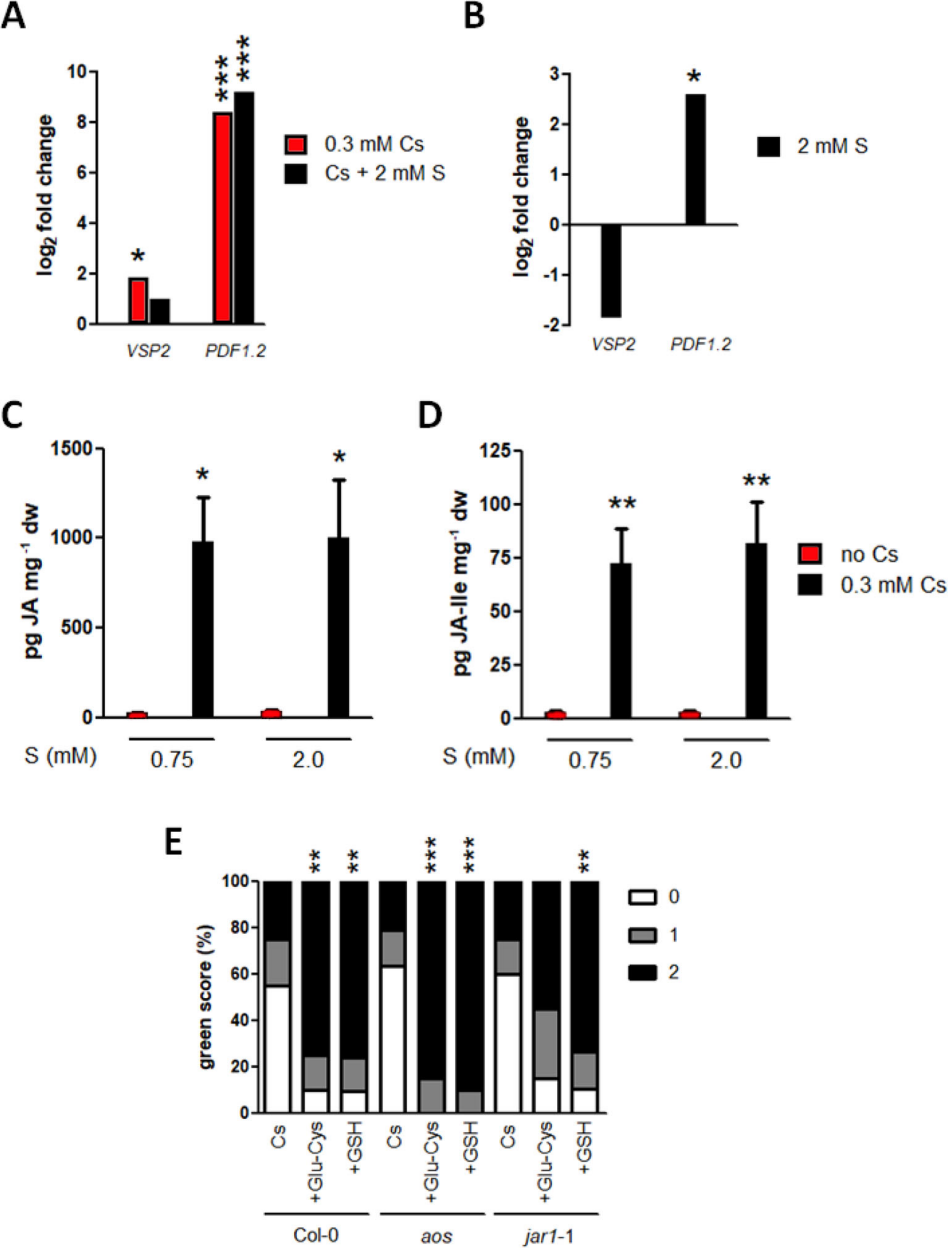
Interaction of cesium stress alleviation by sulphur with jasmonate biosynthesis and signalling. **(A)** Expression of *VSP2* and *PDF1.2* in the wild type (Col-0) grown on media containing 1.75 mM potassium (K) and 0.3 mM cesium (Cs) with or without 2 mM sulphate (S) for eight days. Values are log_2_ ratios relative to expression in the control seedlings grown in the absence of cesium (1.75 mM K + 0.75 mM S). Statistically significant differences were determined by one-way ANOVA with Bonferroni’s multiple comparison posttest (n = 3, * for *P* < 0.05, *** for *P* < 0.001). **(B)** Expression of *VSP2* and *PDF1.2* in Col-0 grown on media containing 1.75 mM K and 2 mM S for eight days. Values are log_2_ ratios relative to expression in the control seedlings grown in the basal S condition (0.75 mM). A statistically significant difference was determined by t-test and marked with an asterisk (n = 3, *P* < 0.05). **(C)** Jasmonic acid (JA) and **(D)** Jasmonyl-isoleucine (JA-Ile) concentrations in Col-0 grown on media containing 0.5 mM K with or without 0.3 mM Cs and 2 mM S for eight days. Statistically significant differences were determined by one-way ANOVA with Bonferroni’s multiple comparison posttest (n = 6, * for *P* < 0.05, ** for *P* < 0.01 relative to the control seedlings grown in the basal S condition without Cs) and error bars represent the SE. **(E)** Percentile green scores for Col-0, *aos* and *jar1*-1 grown on media containing 0.5 mM K and 0.3 mM Cs with or without 100 μM γ-glutamylcysteine (γ-Glu-Cys) or glutathione (GSH) for eight days. Statistically significant differences relative to the control cesium condition (0.5 mM K + 0.75 mM S + 0.3 mM Cs) for each line were determined by Kruskal-Wallis test with Dunn’s multiple comparison posttest (n > 19, ** for *P* < 0.01, *** for *P* < 0.001). No statistical difference was observed between Col-0 and the mutants of the same treatment.

JA-responsive genes, *VSP2* and *PDF1.2*, have previously been confirmed as being induced in response to cesium (Adams et al., 2013) and improved tolerance to alkaline stresses caused by JA has been linked to the ascorbate GSH cycle and glyoxylase metabolism (Mir et al., 2018). Cesium treatment with or without sulphur supply induced *VSP2* in shoots by 3.6 and 2.0 times, respectively, relative to the non-cesium control but the induction was not statistically significant for the concomitant treatment of cesium and sulphur supply (**Figure 7A**). This reduced level of *VSP2* during concomitant treatment could be due to the reduction of *VSP2* by sulphur supply alone (0.28 times) although it was not statistically significant (**Figure 7B**). On the other hand, *PDF1.2* was dramatically up-regulated in shoots treated with cesium alone or together with sulphur supply (333 and 576 times induction, respectively, **Figure 7A**). *PDF1.2* was found to be induced by 6.0 times in response to sulphur supply alone also (**Figure 7B**). There was no statistical difference between cesium treatments alone and together with sulphur supply for expression of both *VSP2* and *PDF1.2*. Consistently, internal concentrations of jasmonic acid and jasmonyl–isoleucine were markedly increased in response to cesium, and sulphur supply did not alter these levels (**Figures 7C, D**). The expression patterns of GSH metabolism-related genes, such as glyoxylase1 (At1g11840), glyoxylase4 (At1g15380), and glutathione-S-transferase24 (At1g17170) were analyzed in response to 0.3 mM cesium with or without 2 mM sulphur supply. However, expression of these genes was not altered by Cs treatment (**Supplementary Figure S2**). JA biosynthesis mutants defective in allene oxide synthase, *aos* (Park et al., 2002), and jasmonate-amido synthetase, *jar1*-1 (Staswick et al., 1992), were also analyzed for cesium response and recovery by GSH. Green scores for *aos* and *jar1*-1 treated with cesium in the absence of sulphur supply were comparable to those of the wild type. Recovery from the inhibitory effects of cesium was observed by addition of γ-Glu-Cys or GSH for both of the mutants, although the degree of recovery for *jar1*-1 by γ-Glu-Cys was somewhat compromised (**Figure 7E**).

## Discussion

Successful phytoremediation relies upon efficient absorption of the contaminant while at the same time maintaining vigorous plant growth, which means tolerance to the contaminant. Radiocesium contamination in soils poses a serious risk to humans and the environment but while potassium supply, a conventional method, is known to recover the healthy growth of the plants exposed to high concentrations of cesium, it causes a reduction in cesium accumulation in the plants, which is not helpful in terms of soil decontamination. Here we report that sulphur supply alleviates cesium-induced growth inhibition without compromising its accumulation in the plants. Increasing the basal level of sulphate (0.75 mM) up to 1.5 mM was not sufficient to reverse the cesium effects but up to 2 mM significantly recovered growth retardation caused by cesium (**Figure 1**). Increased potassium supply was demonstrated to confer “seeming” cesium tolerance by reducing cesium accumulation as well as reducing the degree of cesium-induced potassium loss, in turn resulting in lower cesium/potassium ratios. By contrast, with an abundant supply of sulphur, cesium and potassium concentrations were not altered, suggesting that sulphur supply enhanced “true” tolerance to cesium in the plants. This observation was reinforced by gene expression analysis of a potassium deficiency marker gene, *HAK5*, cesium-induce expression of which was not decreased by sulphur supply, indicating that the degree of potassium deficiency perceived by the plants was not eased. Similarly, sodium stress was alleviated by sulphur supply without altering sodium or potassium concentrations in the plants (**Figure 2**). Improved salt tolerance achieved by high levels of sulphur supplementation has previously been reported in soil-grown mustard but sodium accumulation was shown as being reduced by 100–200 mg sulphur kg^−1^ soil in these cases (Fatma et al., 2014; Fatma et al., 2016). Both cesium and sodium are known to compete with potassium and to initiate a potassium deficiency response in plants, however, sulphur supply did not alleviate potassium deficiency stress. These results suggest that an addition of sulphur somehow helps plants acquire tolerance to the toxic metals but cannot reverse a lack of an essential nutrient.

Since sulphur supply-induced alleviation of cesium stress is not due to reduced levels of cesium or increased levels of potassium, expression of *SULTRs* was tested. Expression of genes encoding high-affinity sulphate transporters in roots, SULTR1;1 and SUTLR1;2, is known to be induced by sulphate starvation (Takahashi et al., 2000; Yoshimoto et al., 2002). These transcripts have also been shown to be increased in response to cadmium and sodium stress (Cao et al., 2014; Yamaguchi et al., 2016; Ferri et al., 2017). However, *SULTR1;1* and *SULTR1;2* were not induced in response to cesium stress but *SULTR1;2* was rather reduced by cesium treatment in the presence of sulphur supply in the optimal potassium condition (**Figure 3**). A low-affinity sulphate transporter gene, *SULTR2;2*, known to be low sulphate-inducible (Takahashi et al., 2000) was also not up-regulated in response to cesium stress. Instead, *SULTR3;5* was mildly induced in response to cesium stress. SULTR3;5 has been reported as being responsible for root-to-shoot transport of sulphate only in the presence of a low-affinity sulphate transporter SULTR2;1 (Kataoka et al., 2004a), although our data indicated no altered expression for *SULTR2;1* in response to cesium (**Supplementary Figure S1**). Mutants for each of the *SULTR* genes were analyzed for cesium response but no phenotypic difference was observed relative to the wild type (**Figure 3**). There was a tendency for higher accumulation of potassium and cesium in *sultr4;1* and *sultr4;2* mutants although potassium accumulation was not statistically significant. SULTR4;1 and SULTR4;2 have been shown to facilitate sulphate efflux from the vacuoles to maintain optimal cellular concentrations of sulphate and the *sultr4;1 sultr4;2* double mutant demonstrates over-accumulation of sulphate in roots (Kataoka et al., 2004b). It is possible that this higher accumulation of sulphate in the mutants promoted higher accumulation of cesium and potassium in our system. Overall, SULTRs did not appear to participate much in cesium stress alleviation, unlike other metal stress response. By contrast, *SULTR1;2*, *SULTR3;1,* and *SULTR3;5* were found to be induced in response to potassium deficiency although none of the single mutants for these genes or any of the other *SULTR* genes showed altered response to potassium deficiency (**Supplementary Figure S1**). This transcriptional induction could be due to a general nutrient deficiency response where deficiency of a nutrient triggers transport/metabolism/signalling of another nutrient (Schachtman and Shin, 2007).

Our results highlight that SULTRs and altered sulphate uptake are unlikely to be involved in alleviation of cesium stress, suggesting that it is the downstream metabolic pathways that are important. Various sulphur metabolism genes have previously been indicated as being induced in response to heavy metal stress or sodium stress (Xiang and Oliver, 1998; Howarth et al., 2003; Tamaoki et al., 2008; Zhu et al., 2016), however, expression of *SERAT2;1*, *OASTLa* and *GSH1* was not altered either in roots or shoots in response to cesium stress (**Figure 4**). In contrast, expression of a GSH synthetase gene, *GSH2*, was mildly induced in response to cesium and concomitantly with sulphur supply suggesting an accumulation of GSH. PCs are a common detoxification agent for heavy metals and PC synthase genes, *PCS1* and *PCS2*, have been shown to be induced by chromium and lead (Fang et al., 2016; Zhu et al., 2016). Under cesium stress, however, these transcripts were not induced but *PCS1* was reduced in shoots with or without sulphur supply instead. Indeed, an increase of PC levels were not detected in response to cesium. This point was reinforced by observation of *cad1*-3 mutant carrying a point mutation in *PCS1* which makes plants sensitive to cadmium (Howden et al., 1995). The degree of cesium response in *cad1*-3 was comparable to that of the wild type (**Figure 5**), indicating that PCs are not important for alleviation of cesium stress. As GSH is the direct precursor of PCs and PCS1 is the major form of PC synthetase (Blum et al., 2010), our results further suggest increased accumulation of GSH in cesium-treated plants. An increase of GSH levels has been reported in plants exposed to sodium, cadmium, chromium and zinc (Nocito et al., 2006; Fatma et al., 2014; Nazar et al., 2014; Fang et al., 2016; Per et al., 2016). In agreement with our hypothesis, GSH levels were increased in the plants suffering from cesium stress and exogenous application of GSH to the cesium-exposed plants could alleviate the growth retardation effects of cesium to the same degree as with sulphur supply (**Figure 5**). Moreover, *cad1*-3 displayed a wild type-like response to GSH-induced alleviation of cesium effects, confirming that this alleviation event is dependent on GSH but independent of PCs. Consistently, treatments with concomitant supplementation of Glu and Cys together (or their synthetic product, γ-Glu-Cys) with or without Gly, which lead to GSH synthesis, could alleviate cesium stress to the level achieved through sulphur supply (**Figure 6**). Accumulation of γ-Glu-Cys has been demonstrated to inhibit plant growth in a JA-dependent manner (Wei et al., 2015) but this growth inhibition did not affect the alleviation effects of γ-Glu-Cys for cesium. An addition of Gly attenuated this growth inhibitory effects of γ-Glu-Cys possibly through pushing the reaction forward towards the direction of GSH synthesis so that γ-Glu-Cys was not accumulated. By contrast, the alleviation effects of single supplementation of Glu, Cys or Gly were observed but rather limited (**Figure 6**). To our surprise, and intriguingly in a way, inhibition of GSH synthesis did not cancel the alleviation effects of GSH precursors. As a mutation in *GSH2* causes seedling lethality (*gsh2*-T, according to the description provided by the ABRC), a specific inhibitor of the function of GSH1 (Griffith, 1982), BSO, was employed. BSO-treated plants cannot synthesize Glu and Cys into γ-Glu-Cys and decreased accumulation of GSH was observed in the seedlings treated with cesium and BSO (**Figure 5**). In theory, a concomitant treatment with Glu, Cys and BSO should not provide an alleviation effect if GSH was the sole alleviator of cesium stress, but this was not the case. Besides, BSO treatment alone somewhat alleviated cesium stress although it was not statistically significant. These findings strongly suggest that not only GSH but also GSH precursors, when concomitantly treated, have their own alleviatory function or feed into the pathways other than GSH synthesis which produce alleviatory effects against cesium stress. Our previous metabolic profiling has revealed a marked increase of Cys in both roots and shoots, as well as Glu and Gly to a lesser extent in shoots, in cesium-treated plants (Adams et al., 2017). Cys and its derivative, methyl cysteinate, were also demonstrated to be capable of chelating with cesium and functioning as cesium accumulators in plants (Adams et al., 2017). A dramatic increase of Cys in response to cesium was confirmed in the current study but interestingly, BSO treatment did not further increase Cys (**Figure 5**), suggesting that accumulated Cys was quickly turned into other metabolites which might also have an alleviation effect. Together, it can be speculated that plants exposed to cesium accumulate GSH and its biosynthetic intermediates as a detoxification measure to protect the cells from the deleterious effects of cesium and exogenous application of sulphur-containing compounds can help the plants better perform this procedure.

We have previously shown that JA responsive genes, *VSP2* and *PDF1.2*, are induced in response to cesium stress and JA biosynthesis and signalling mutants, *aos* and *coi1*-16, display increased tolerance against cesium in terms of root growth inhibition (Adams et al., 2013). This indicates that cesium inhibits root growth through JA biosynthesis and signalling pathways. In order to test whether sulphur supply could turn down the JA pathways, JAs were quantified. Treatment with cesium dramatically increased JA and JA-Ile accumulation in the plants and consistently induced *VSP2* and *PDF1.2* expression (**Figure 7**). Sulphur supply neither decreased accumulation of JAs nor reduced expression of JA responsive genes, suggesting that sulphur supply does not alleviate cesium stress by reducing JA production. Interaction between JA pathways and sulphur metabolism has been documented in certain aspects: a positive regulator and a key transcription factor of JA signalling, MYC2, activates *GSH1 via* direct binding on its promoter (Yuan et al., 2017) and GSH is the key regulator of JA pathway (Mhamdi et al., 2010; Han et al., 2013). Exogenous application of JAs induces a series of sulphur metabolism genes including *GSH1* and *GSH2* and increases Cys and GSH levels in the plants (Jost et al., 2005; Sasaki-Sekimoto et al., 2005). Conversely, GSH application induces a wide variety of JA synthesis and signalling genes (Han et al., 2013; Hacham et al., 2014; Cheng et al., 2015). However, jasmonate biosynthesis mutants, *aos* and *jar1*-1, showed a wild type-like aerial phenotype to cesium and recovery from cesium stress by GSH and γ-Glu-Cys albeit to a lesser extent for *jar1*-1 by γ-Glu-Cys (**Figure 7**). These findings imply that cesium stress alleviation by GSH and its precursors does not rely on alteration in JA synthesis and, at the same time, is not disrupted by inability of JA synthesis.

**Figure 8:**
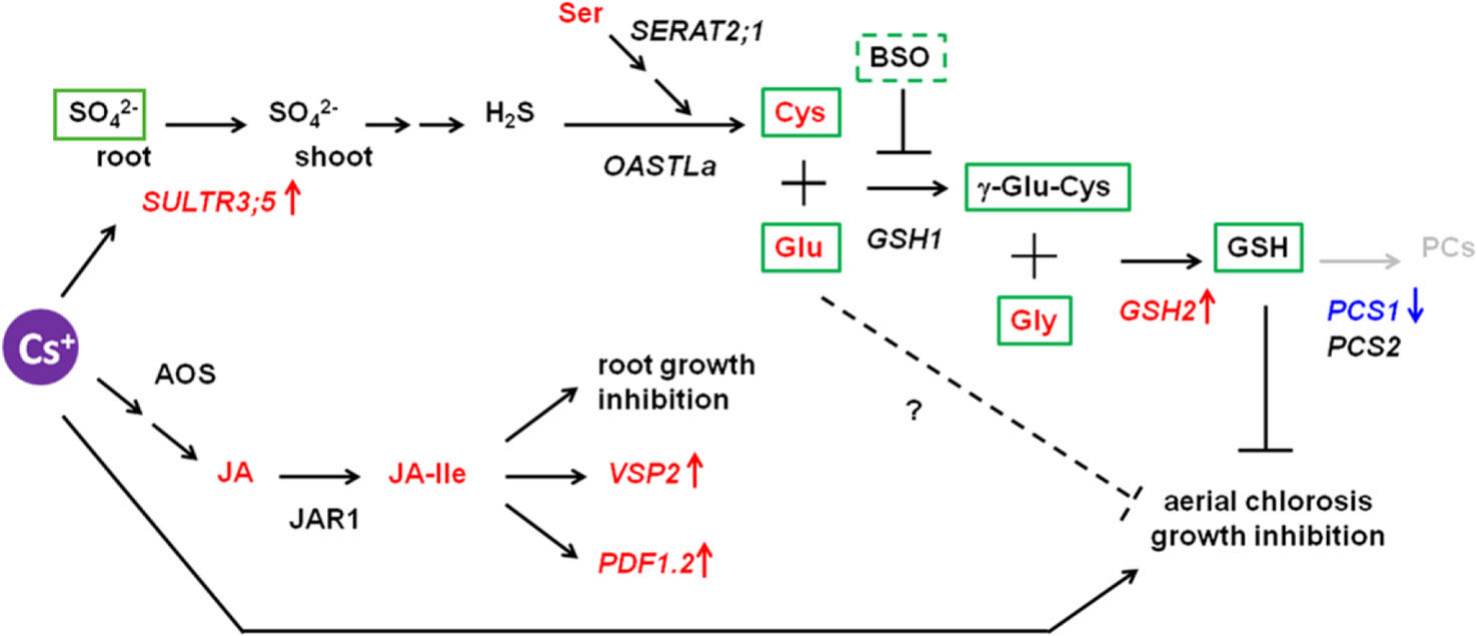
Proposed model for interaction between cesium response and sulphur-mediated alleviation. The genes denoted in italics are the ones whose expression was investigated in this study. The transcripts and the metabolites indicated in red and blue were shown in this study or the previous study (Adams et al., 2017) to be increased or decreased, respectively, in response to cesium. The metabolites in green boxes are the ones with an ability to alleviate cesium-induced aerial chlorosis. The pathway in grey was proposed not to be involved.

Taken all together, GSH and its biosynthetic intermediates, but not PCs, have a capacity to mitigate cesium stress and this mitigation is independent of alteration in potassium/cesium accumulation. A predicted model is given in **Figure 8**. Plants exposed to cesium possibly promote root-to-shoot transport of sulphate by the function of SULTR3;5, accumulate Cys as well as Glu and Gly and increase GSH accumulation through induction of *GSH2* and reduction of *PCS1* in shoots to overcome cesium-induced aerial chlorosis and growth retardation. GSH biosynthetic intermediates also have alleviatory effects against cesium as they are or in as-yet-unknown pathways. It is well known that an increase of GSH helps to detoxify the reactive oxygen species (ROS) which were elevated by various abiotic stresses including alkaline metal stresses (Choe et al., 2013). It is possible that alleviation effects of cesium stress by GSH or Cys results from their thiol residues which have a potential to reduce ROS caused by Cs stress. However, our preliminary observation could not recognize ROS accumulation in response to cesium while ROS accumulation was clearly observed in the plants experiencing potassium deficiency (**Supplementary Figure S3**) as described before (Shin and Schachtman, 2004). As sulphur supply alleviates sodium stress but not potassium deficiency stress, this alleviation is not due to the recovery effects against a general nutrition deficiency by another nutrition supply but potentially due to the detoxification effects against xenobiotics. GSH is also known to conjugate with xenobiotics including toxic metals with the aid of GSH *S*-transferases (GSTs) and to sequestrate them into the vacuoles (Dixon et al., 2002; Hasanuzzaman et al., 2017). As GSTs constitute a large gene family in Arabidopsis, it is intriguing to investigate whether cesium is conjugated with GSH and detoxified by one of the GSTs. It would also be interesting to reveal the mechanism by which GSH biosynthetic precursors alleviate cesium stress independently of GSH biosynthesis.

## Data Availability Statement

No datasets was generated or analyzed for this study.

## Author Contributions

EA, TM, SW and NO-O did experiments. EA, MS and RS designed the experiments. EA and RS wrote the manuscript.

## Conflict of Interest

The authors declare that the research was conducted in the absence of any commercial or financial relationships that could be construed as a potential conflict of interest.

## Abbreviations

BSO, buthionine sulphoximine; Cys, cysteine; γ-Glu-Cys, γ-glutamylcysteine; Glu, glutamic acid; Gly, glycine; GSH, glutathione; JA, jasmonate/jasmonic acid; JA-Ile, jasmonyl-isoleucine; PC, phytochelatin; ROS, reactive oxygen species; Ser, serine.

## References

[B1] Adams E. Abdollahi P. Shin R. (2013). Cesium inhibits plant growth through jasmonate signaling in *Arabidopsis thaliana* . Int. J. Mol. Sci. 14, 4545–4559. 10.3390/ijms14034545 23439557 PMC3634425

[B2] Adams E. Miyazaki T. Hayaishi-Satoh A. Han M. Kusano M. Khandelia H. (2017). A novel role for methyl cysteinate, a cysteine derivative, in cesium accumulation in *Arabidopsis thaliana* . Sci. Rep. 7, 43170. 10.1038/srep43170 28230101 PMC5322390

[B3] Adams E. Miyazaki T. Saito S. Uozumi N. Shin R. (2018). Cesium inhibits plant growth primarily through reduction of potassium influx and accumulation in Arabidopsis. Plant Cell Physiol. 60, 63–76. 10.1093/pcp/pcy188. 30219884

[B4] Anjum N. A. Gill R. Kaushik M. Hasanuzzaman M. Pereira E. Ahmad I. (2015). ATP-sulfurylase, sulfur-compounds, and plant stress tolerance. Front. Plant Sci. 6, 210. 10.3389/fpls.2015.00210 25904923 PMC4387935

[B5] Blum R. Meyer K. C. Wunschmann J. Lendzian K. J. Grill E. (2010). Cytosolic action of phytochelatin synthase. Plant Physiol. 153, 159–169. 10.1104/pp.109.149922 20304971 PMC2862410

[B6] Burger A. Lichtscheidl I. (2018). Stable and radioactive cesium: a review about distribution in the environment, uptake and translocation in plants, plant reactions and plants’ potential for bioremediation. Sci. Total Environ. 618, 1459–1485. 10.1016/j.scitotenv.2017.09.298 29122347

[B7] Cao M. J. Wang Z. Zhao Q. Mao J. L. Speiser A. Wirtz M. (2014). Sulfate availability affects ABA levels and germination response to ABA and salt stress in *Arabidopsis thaliana* . Plant J. 77, 604–615. 10.1111/tpj.12407. 24330104

[B8] Cazale A. C. Clemens S. (2001). *Arabidopsis thaliana* expresses a second functional phytochelatin synthase. FEBS Lett. 507, 215–219. 11684101 10.1016/s0014-5793(01)02976-3

[B9] Cheng M. C. Ko K. Chang W. L. Kuo W. C. Chen G. H. Lin T. P. (2015). Increased glutathione contributes to stress tolerance and global translational changes in Arabidopsis. Plant J. 83, 926–939. 10.1111/tpj.12940. 26213235

[B10] Choe Y.-H. Kim Y.-S. Kim I.-S. Bae M.-J. Lee E.-J. Kim Y.-H. (2013). Homologous expression of γ-glutamylcysteine synthetase increases grain yield and tolerance of transgenic rice plants to environmental stresses. J. Plant Physiol. 170, 610–618. 10.1016/j.jplph.2012.12.002 23294545

[B11] Dixon D. P. Lapthorn A. Edwards R. (2002). Plant glutathione transferases. Genome Biol. 3, 3004. 10.1186/gb-2002-3-3-reviews3004 PMC139027 11897031

[B12] Dushenkov S. (2003). Trends in phytoremediation of radionuclides. Plant Soil 249, 167–175.

[B13] Fang H. Liu Z. Jin Z. Zhang L. Liu D. Pei Y. (2016). An emphasis of hydrogen sulfide-cysteine cycle on enhancing the tolerance to chromium stress in *Arabidopsis* . Environ. Pollut. 213, 870–877. 10.1016/j.envpol.2016.03.035 27038574

[B14] Fatma M. Asgher M. Masood A. Khan N. A. (2014). Excess sulfur supplementation improves photosynthesis and growth in mustard under salt stress through increased production of glutathione. Environ. Exp. Bot. 107, 55–63.

[B15] Fatma M. Masood A. Per T. S. Khan N. A. (2016). Nitric oxide alleviates salt stress inhibited photosynthetic performance by interacting with sulfur assimilation in mustard. Front. Plant Sci. 7, 521. 10.3389/fpls.2016.00521 27200007 PMC4842777

[B16] Ferri A. Lancilli C. Maghrebi M. Lucchini G. Sacchi G. A. Nocito F. F. (2017). The sulfate supply maximizing Arabidopsis shoot growth is higher under long- than short-term exposure to cadmium. Front. Plant Sci. 8, 854. 10.3389/fpls.2017.00854 28588602 PMC5439006

[B17] Griffith O. W. (1982). Mechanism of action, metabolism, and toxicity of buthionine sulfoximine and its higher homologs, potent inhibitors of glutathione synthesis. J. Biol. Chem. 257, 13704–13712. 6128339

[B18] Hacham Y. Koussevitzky S. Kirma M. Amir R. (2014). Glutathione application affects the transcript profile of genes in *Arabidopsis* seedling. J. Plant Physiol. 171, 1444–1451. 10.1016/j.jplph.2014.06.016 25077999

[B19] Hampton C. R. Bowen H. C. Broadley M. R. Hammond J. P. Mead A. Payne K. A. (2004). Cesium toxicity in *Arabidopsis* . Plant Physiol. 136, 3824–3837. 15489280 10.1104/pp.104.046672 PMC527179

[B20] Han Y. Mhamdi A. Chaouch S. Noctor G. (2013). Regulation of basal and oxidative stress-triggered jasmonic acid-related gene expression by glutathione. Plant Cell Environ. 36, 1135–1146. 10.1111/pce.12048 23210597

[B21] Hasanuzzaman M. Nahar K. Anee T. I. Fujita M. (2017). Glutathione in plants: biosynthesis and physiological role in environmental stress tolerance. Physiol. Mol. Biol. Plants 23, 249–268. 10.1007/s12298-017-0422-2 28461715 PMC5391355

[B22] Hatzfeld Y. Maruyama A. Schmidt A. Noji M. Ishizawa K. Saito K. (2000). β-Cyanoalanine synthase is a mitochondrial cysteine synthase-like protein in spinach and Arabidopsis. Plant Physiol. 123, 1163–1171. 10889265 10.1104/pp.123.3.1163 PMC59079

[B23] Howarth J. R. Dominguez-Solis J. R. Gutierrez-Alcala G. Wray J. L. Romero L. C. Gotor C. (2003). The serine acetyltransferase gene family in *Arabidopsis thaliana* and the regulation of its expression by cadmium. Plant Mol. Biol. 51, 589–598. 10.1023/A:1022349623951 12650624

[B24] Howden R. Goldsbrough P. B. Andersen C. R. Cobbett C. S. (1995). Cadmium-sensitive, *cad1* mutants of *Arabidopsis thaliana* are phytochelatin deficient. Plant Physiol. 107, 1059–1066. 7770517 10.1104/pp.107.4.1059 PMC157237

[B26] Jost R. Berkowitz O. Wirtz M. Hopkins L. Hawkesford M. J. Hell R. (2000). Genomic and functional characterization of the *oas* gene family encoding O-acetylserine (thiol) lyases, enzymes catalyzing the final step in cysteine biosynthesis in *Arabidopsis thaliana* . Gene 253, 237–247. 10940562 10.1016/s0378-1119(00)00261-4

[B25] Jost R. Altschmied L. Bloem E. Bogs J. Gershenzon J. Hahnel U. (2005). Expression profiling of metabolic genes in response to methyl jasmonate reveals regulation of genes of primary and secondary sulfur-related pathways in *Arabidopsis thaliana* . Photosyn. Res. 86, 491–508. 10.1007/s11120-005-7386-8 16307302

[B27] Jung I. L. Ryu M. Cho S. K. Shah P. Lee J. H. Bae H. (2015). Cesium toxicity alters microRNA processing and AGO1 expressions in *Arabidopsis thaliana* . PloS One 10, e0125514. 10.1371/journal.pone.0125514 25946015 PMC4422737

[B28] Kanno Y. Oikawa T. Chiba Y. Ishimaru Y. Shimizu T. Sano N. (2016). AtSWEET13 and AtSWEET14 regulate gibberellin-mediated physiological processes. Nat. Commun. 7, 13245. 10.1038/ncomms13245 27782132 PMC5095183

[B29] Kataoka T. Hayashi N. Yamaya T. Takahashi H. (2004a). Root-to-shoot transport of sulfate in Arabidopsis. Evidence for the role of SULTR3;5 as a component of low-affinity sulfate transport system in the root vasculature. Plant Physiol. 136, 4198–4204. 10.1104/pp.104.045625 15531709 PMC535849

[B30] Kataoka T. Watanabe-Takahashi A. Hayashi N. Ohnishi M. Mimura T. Buchner P. (2004b). Vacuolar sulfate transporters are essential determinants controlling internal distribution of sulfate in Arabidopsis. Plant Cell. 16, 2693–2704. 10.1105/tpc.104.023960 15367713 PMC520965

[B31] Kawashima C. G. Berkowitz O. Hell R. Noji M. Saito K. (2005). Characterization and expression analysis of a serine acetyltransferase gene family involved in a key step of the sulfur assimilation pathway in *Arabidopsis* . Plant Physiol. 137, 220–230. 10.1104/pp.104.045377 15579666 PMC548853

[B33] May M. J. Leaver C. J. (1994). *Arabidopsis thaliana* γ-glutamylcysteine synthetase is structurally unrelated to mammalian, yeast, and *Escherichia coli* homologs. Proc. Nat. Acad. Sci. U.S.A. 91, 10059–10063. 10.1073/pnas.91.21.10059 PMC44957 7937837

[B32] Mhamdi A. Hager J. Chaouch S. Queval G. Han Y. Taconnat L. (2010). Arabidopsis GLUTATIONE REDUCTASE1 plays a crucial role in leaf responses to intracellular hydorgen peroxide and in ensuring appropiate gene expression through both salicylic acid and jasmonic acid signaling pathways. Plant Physiol. 153, 1144–1160. 10.1104/pp.110.153767 20488891 PMC2899936

[B34] Minocha R. Thangavel P. Dhankher O. P. Long S. (2008). Separation and quantification of monothiols and phytochelatins from a wide variety of cell cultures and tissues of trees and other plants using high performance liquid chromatography. J. Chromatogr. A. 1207, 72–83. 10.1016/j.chroma.2008.08.023 18760414

[B35] Mir M. A. John R. Alyemeni M. N. Alam P. Ahmad P. (2018). Jasmonic acid ameliorates alkaline stress by improving gorwth peformance, ascorbate glutathione cycle and glyoxylase system in mainze seedlings. Sci. Rep. 8, 2831. 10.1038/s41598-018-21097-3 29434207 PMC5809373

[B36] Nazar R. Khan M. I. Iqbal N. Masood A. Khan N. A. (2014). Involvement of ethylene in reversal of salt-inhibited photosynthesis by sulfur in mustard. Physiol. Plant 152, 331–344. 10.1111/ppl.12173 24547902

[B37] Nishida S. Duan G. L. Ohkama-Ohtsu N. Uraguchi S. Fujiwara T. (2016). Enhanced arsenic sensitivity with excess phytochelatin accumulation in shoots of a SULTR1;2 knockout mutant of *Arabidopsis thaliana* (L.) Heynh. Soil Sci. Plant Nutr. 62, 367–372. 10.1080/00380768.2016.1150790

[B38] Nocito F. F. Lancilli C. Crema B. Fourcroy P. Davidian J. C. Sacchi G. A. (2006). Heavy metal stress and sulfate uptake in maize roots. Plant Physiol. 141, 1138–1148. 10.1104/pp.105.076240 16698905 PMC1489904

[B39] Noctor G. Queval G. Mhamdi A. Chaouch S. Flyer C. H. (2011). “Glutathione,” in The Arabidopsis Book, e0142. 10.1199/tab0142 PMC3267239 22303267

[B40] Park J. H. Halitschke R. Kim H. B. Baldwin I. T. Feldmann K. A. Feyereisen R. (2002). A knock-out mutation in allene oxide synthase results in male sterility and defective wound signal transduction in *Arabidopsis* due to a block in jasmonic acid biosynthesis. Plant J. 31, 1–12. 12100478 10.1046/j.1365-313x.2002.01328.x

[B41] Per T. S. Khan N. A. Masood A. Fatma M. (2016). Methyl jasmonate alleviates cadmium-induced photosynthetic damages through increased S-assimilation and glutathione production in mustard. Front. Plant Sci. 7, 1933. 10.3389/fpls.2016.01933 28066485 PMC5177644

[B42] Sahr T. Voigt G. Paretzke H. G. Schramel P. Ernst D. (2005a). Caesium-affected gene expression in *Arabidopsis thaliana* . New Phytol. 165, 747–754. 10.1111/j.1469-8137.2004.01282.x 15720685

[B43] Sahr T. Voigt G. Schimmack W. Paretzke H. G. Ernst D. (2005b). Low-level radiocaesium exposure alters gene expression in roots of Arabidopsis. New Phytol. 168, 141–148. 10.1111/j.1469-8137.2005.01485.x 16159328

[B44] Sasaki-Sekimoto Y. Taki N. Obayashi T. Aono M. Matsumoto F. Sakurai N. (2005). Coordinated activation of metabolic pathways for antioxidants and defence compounds by jasmonates and their roles in stress tolerance in *Arabidopsis* . Plant J. 44, 653–668. 16262714 10.1111/j.1365-313X.2005.02560.x

[B45] Schachtman D. P. Shin R. (2007). Nutrient sensing and signaling: NPKS. Ann. Rev. Plant Biol. 58, 47–69. 17067284 10.1146/annurev.arplant.58.032806.103750

[B46] Schneider C. A. Rasband W. S. Eliceiri K. W. (2012). NIH Image to ImageJ: 25 years of image analysis. Nat. Methods 9, 671–675. 22930834 10.1038/nmeth.2089 PMC5554542

[B47] Seth C. S. Remans T. Keunen E. Jozefczak M. Gielen H. Opdenakker K. (2012). Phytoextraction of toxic metals: a central role for glutathione. Plant Cell Environ. 35, 334–346. 10.1111/j.1365-3040.2011.02338.x 21486307

[B48] Shin R. Schachtman D. P. (2004). Hydrogen peroxide mediates plant root cell response to nutrient deprivation. Proc. Nat. Acad. Sci. U.S.A. 101, 8827–8832. 10.1073/pnas.0401707101 PMC423280 15173595

[B49] Staswick P. E. Su W. P. Howell S. H. (1992). Methyl jasmonate inhibition of root growth and induction of a leaf protein are decreased in an *Arabidopsis thaliana* mutant. Proc. Nat. Acad. Sci. U.S.A. 89, 6837–6840. 10.1073/pnas.89.15.6837 PMC49599 11607311

[B50] Takahashi H. Watanabe-Takahashi A. Smith F. W. Blake-Kalff M. Hawkesford M. J. Saito K. (2000). The roles of three functional sulphate transporters involved in uptake and translocation of sulphate in *Arabidopsis thaliana* . Plant J. 23, 171–182. 10929111 10.1046/j.1365-313x.2000.00768.x

[B51] Tamaoki M. Freeman J. L. Pilon-Smits E. A. (2008). Cooperative ethylene and jasmonic acid signaling regulates selenite resistance in *Arabidopsis* . Plant Physiol. 146, 1219–1230. 10.1104/pp.107.110742 18178671 PMC2259059

[B52] Ullmann P. Gondet L. Potier S. Bach T. J. (1996). Cloning of *Arabidopsis thaliana* glutathione synthetase (GSH2) by functional complementation of a yeast *gsh2* mutant. Eur. J. Biochem. 236, 662–669. 8612643 10.1111/j.1432-1033.1996.00662.x

[B53] Wei H. H. Rowe M. Riethoven J. J. M. Grove R. Adamec J. Jikumaru Y. (2015). Overaccumulation of γ-glutamylcysteine in a jasmonate-hypersensitive *Arabidopsis* mutant causes jasmonate-dependent growth inhibition. Plant Physiol. 169, 1371–1381. 10.1104/pp.15.00999 26282239 PMC4587470

[B54] White P. J. Broadley M. R. (2000). Mechanisms of caesium uptake by plants. New Phytol. 147, 241–256.

[B55] Xiang C. Oliver D. J. (1998). Glutathione metabolic genes coordinately respond to heavy metals and jasmonic acid in Arabidopsis. Plant Cell. 10, 1539–1550. 9724699 10.1105/tpc.10.9.1539 PMC144077

[B56] Yamaguchi C. Takimoto Y. Ohkama-Ohtsu N. Hokura A. Shinano T. Nakamura T. (2016). Effects of cadmium treatment on the uptake and translocation of sulfate in *Arabidopsis thaliana* . Plant Cell Physiol. 57, 2353–2366. 10.1093/pcp/pcw156 27590710

[B57] Yoshimoto N. Takahashi H. Smith F. W. Yamaya T. Saito K. (2002). Two distinct high-affinity sulfate transporters with different inducibilities mediate uptake of sulfate in *Arabidopsis* roots. Plant J. 29, 465–473. 11846879 10.1046/j.0960-7412.2001.01231.x

[B58] Yuan L. B. Dai Y. S. Xie L. J. Yu L. J. Zhou Y. Lai Y. X. (2017). Jasmonate regulates plant responses to postsubmergence reoxygenation through transcriptional activation of antioxidant synthesis. Plant Physiol. 173, 1864–1880. 10.1104/pp.16.01803 28082717 PMC5338657

[B60] Zhu Y. G. Smolders E. (2000). Plant uptake of radiocaesium: a review of mechanisms, regulation and application. J. Exp. Bot. 51, 1635–1645. 10.1093/jexbot/51.351.1635 11053452

[B59] Zhu F. Y. Chan W. L. Chen M. X. Kong R. P. Cai C. Wang Q. (2016). SWATH-MS quantitative proteomic investigation reveals a role of jasmonic acid during lead response in *Arabidopsis* . J. Proteome Res. 15, 3528–3539. 10.1021/acs.jproteome.6b00258 27599093

