# Supplementary material for: Glutathione and Its Biosynthetic Intermediates Alleviate Cesium Stress in Arabidopsis
Source: Front Plant Sci. 2020 Jan 21;10:1711. doi: 10.3389/fpls.2019.01711 (PMC6985154; doi:10.3389/fpls.2019.01711)
Supplement: Supplementary file 1 [file DataSheet_1.pdf]

Supplementary Table S1. Sequences of qRT-PCR primers used in this study

name	sequence
SULTR1;3-f	CCCTAAGTATGGTCTATATTCAAGTTTTGTTCCTC
SULTR1;3-r	GATCTCAGCACGAAGCAGAGTACCTAATAG
SULTR2;1-f	ATGACGGTTAAGACTCCCGGA
SULTR2;1-r	CGACCCATCCCATAATCCTTT
SULTR2;2-f	CGACATGTCTTGCGTGATGGGCG
SULTR2;2-r	GCTCGCTTCAATTTGTGAAGTACCCT
SULTR4;1-f	TGACACCTCCAATGAAGCTCGTCCTC
SULTR4;1-r	AATTGGTGGAAGGCCAGCTAGTTTCG
SULTR4;2-f	GATTCCACCTCTTCTGATCCTCACT
SULTR4;2-r	TATTGGTTGAAGCCCAGCTAACCTT
SERAT2;1-f	GCTCATACCCTCTGGAAACAGAACAGAAAAATC
SERAT2;1-r	GCCCGTCGCATGGTCTAAAAGAATC
OASTLa-f	CTGTATGGAGTGGAGCCAGTTGAAAGTG
OASTLa-r	GATTCATCACTTGAAACCTGAACAACTTCGTCAAT
GSH1-f	TACGCACTCGATGTCCCTATGTACTTTGCCTAC
GSH1-r	TCATCTCCAAGTATCTCTTCAACCGAACCTCTG
GSH2-f	CTCAGAGAGAAGGCGGAGGAAACAACATCTATG
GSH2-r	CCGAGTTCTGATATAGCTTGATGCTTATGGTAAACGC
PCS1-f	CTTCCGCCGAAGGCAAGCTAATCTTCAATG
PCS1-r	AACACCACTGAGAGACTAGCCAAACCAC
PCS2-f	AAATGTATCAAAGGTCTTGGTGAGGAGAAAGTGAC
PCS2-r	GAAGCAAAGTCGGGTGGCTAACCATG
TUB2-f	GCCAATCCGGTGCTGGTAACA
TUB2-r	CATACCAGATCCAGTTCCTCCTCCC

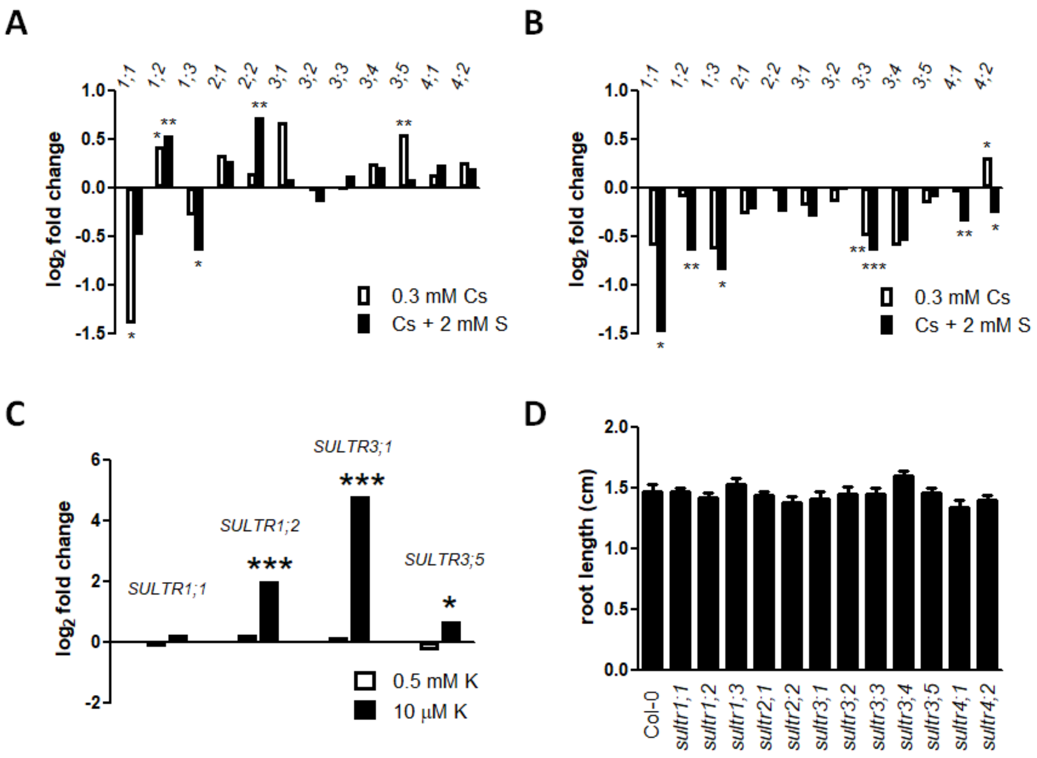

Supplementary Fig. S1. Gene expression of sulphate transporter genes (*SULTR*s) in response to cesium and potassium deficiency stress and phenotype of potassium-deprived *sultr* mutants. Expression of *SULTR*s in roots (A) and shoots (B) of the wild type (Col-0) grown on media containing 0.5 mM potassium (K) and 0.3 mM cesium (Cs) with or without 2 mM sulphate (S) for eight days. Values are log2ratios relative to expression in the control seedlings grown in the absence of cesium. Statistically significant differences were determined by one-way ANOVA with Bonferroni's multiple comparison posttest (n=3, * for P<0.05, ** for P<0.01, *** for P<0.001). (C) Expression of *SULTR*s in roots of Col-0 grown on media containing 10 µM or 0.5 mM K for eight days. Values are log2ratios relative to expression in the optimal potassium condition (1.75 mM). Statistically significant differences were determined by one-way ANOVA with Bonferroni's multiple comparison posttest (n=3, * for P<0.05, *** for P<0.001). (D) Root lengths of *sultr* mutants grown in K deficiency (10 µM) for eight days. No statistical difference relative to Col-0 was determined by one-way ANOVA with Bonferroni's multiple comparison posttest (n>19). Error bars represent the SE.

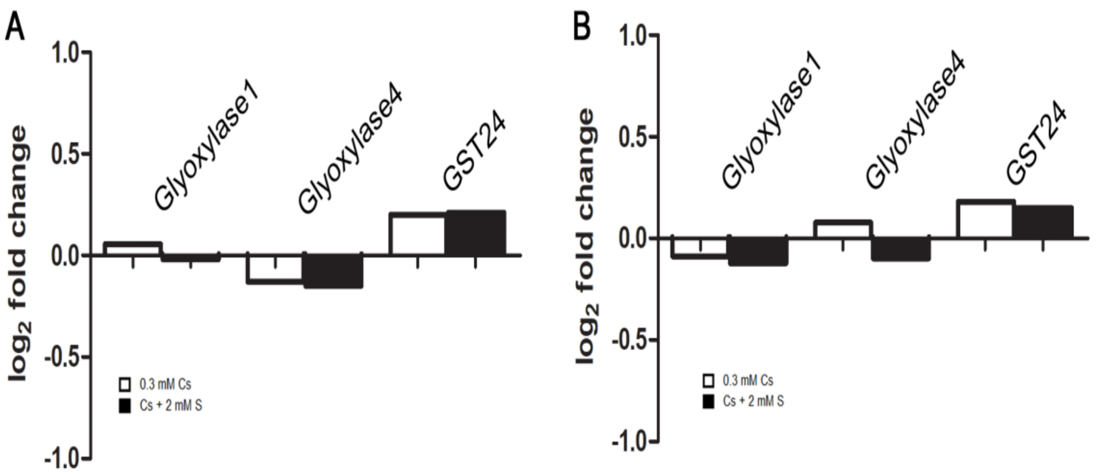

Supplementary Fig. S2. Expression of glyoxylase1 (At1g11840), glyoxylase4 (At1g15380), and glutathione-S-transferase24 (At1g17170) in response to cesium. Gene expression in roots (A) and shoots (B) of the wild type (Col-0) grown on media containing 1.75 mM potassium (K) and 0.3 mM cesium (Cs) with or without 2 mM sulphate (S) for eight days. Values are log2 ratios relative to expression in the control seedlings grown in the absence of cesium (1.75 mM K + 0.75 mM S). No statistical difference was determined by one-way ANOVA with Bonferroni’s multiple comparison posttest.

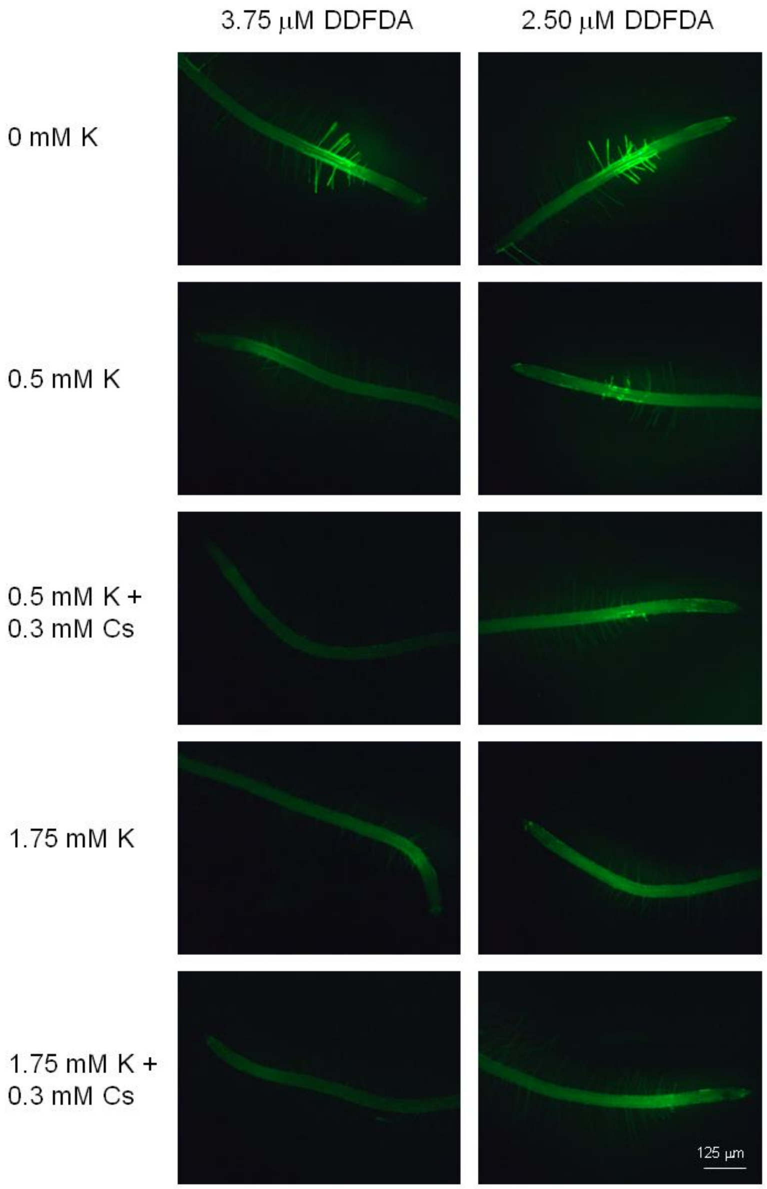

Supplementary Fig. S3. Subcellular images of ROS accumulation in the roots exposed to cesium and potassium deficiency stress. Three-day-old Col-0 seedlings germinated on the indicated concentrations of K and Cs were incubated for approximately 20 hours in liquid media with the same composition without sucrose, then in liquid media containing 5-(and 6-) carboxy-2',7'-difluorodihydrofluorescein diacetate(DFFDA) in the dark for 15 minutes prior to imaging. The scale bar indicates 125 μm. n>10 seedlings.
